# EIGER2 hybrid-photon-counting X-ray detectors for advanced synchrotron diffraction experiments

**DOI:** 10.1107/S160057752300454X

**Published:** 2023-06-21

**Authors:** Tilman Donath, Dubravka Šišak Jung, Max Burian, Valeria Radicci, Pietro Zambon, Andrew N. Fitch, Catherine Dejoie, Bingbing Zhang, Marie Ruat, Michael Hanfland, Cameron M. Kewish, Grant A. van Riessen, Denys Naumenko, Heinz Amenitsch, Gleb Bourenkov, Gerard Bricogne, Ashwin Chari, Clemens Schulze-Briese

**Affiliations:** a DECTRIS Ltd, Täfernweg 1, 5405 Baden, Switzerland; b European Synchrotron Radiation Facility (ESRF), 38043 Grenoble, France; c Institute of High Energy Physics, Chinese Academy of Sciences, 19B Yuquan Road, Beijing 100049, People’s Republic of China; d Australian Synchrotron, Australian Nuclear Science and Technology Organisation (ANSTO), Clayton, Victoria 3168, Australia; e Department of Mathematical and Physical Sciences, School of Computing, Engineering and Mathematical Sciences, La Trobe University, Bundoora, Victoria 3086, Australia; f Institute for Inorganic Chemistry, Graz University of Technology, Stremayrgasse 9, 8010 Graz, Austria; g Hamburg Outstation c/o DESY, European Molecular Biology Laboratory, Notkestrasse 85, 22607 Hamburg, Germany; h Global Phasing Ltd, Sheraton House, Castle Park, Cambridge CB3 0AX, United Kingdom; i Max Planck Institute for Multidisciplinary Sciences, Am Fassberg 11, 37077 Göttingen, Germany; Paul Scherrer Institut, Switzerland

**Keywords:** hybrid photon counting, pixel detector, silicon, cadmium telluride, count rate, quantum efficiency, EIGER2

## Abstract

The ability to utilize a hybrid-photon-counting detector to its full potential can significantly influence data quality, data collection speed, as well as development of elaborate data acquisition schemes. This paper facilitates the optimal use of EIGER2 detectors by providing theory and practical advice on (i) the relation between detector design, technical specifications and operating modes, (ii) the use of corrections and calibrations, and (iii) new acquisition features: a double-gating mode, 8-bit readout mode for increasing temporal resolution, and lines region-of-interest readout mode for frame rates up to 98 kHz. Examples of the implementation and application of EIGER2 at several synchrotron sources (ESRF, PETRA III/DESY, ELETTRA, AS/ANSTO) are presented: high accuracy of high-throughput data in serial crystallography using hard X-rays; suppressing higher harmonics of undulator radiation, improving peak shapes, increasing data collection speed in powder X-ray diffraction; faster ptychography scans; and cleaner and faster pump-and-probe experiments.

## Abstract 2

A presentation of how EIGER2 detectors work and how to best use their newly released features for fast image acquisitions and advanced acquisition schemes.

## 1. Introduction

Unlocking the potential of synchrotron and laboratory X-ray experiments largely relies on continuous detector development. In this context, the development has been pursuing means to enhance the data quality and the speed at which data can be collected. This left a legacy of detector technologies, such as scintillation counters, proportional counters, imaging plates, flat panels and complementary metal-oxide semiconductor active pixel detectors. Hybrid-photon-counting (HPC) technology (Brönnimann & Trüb, 2016 ▸) was added to this list at the end of the 20th century, with the first proof-of-concept for use at synchrotron sources (Manolopoulos *et al.*, 1999 ▸). Ever since, HPC detectors have been impacting almost all X-ray applications at synchrotrons and in laboratories (Förster *et al.*, 2019 ▸). The following paragraphs summarize how this impact is related to features of HPC technology.

**Figure 1:**
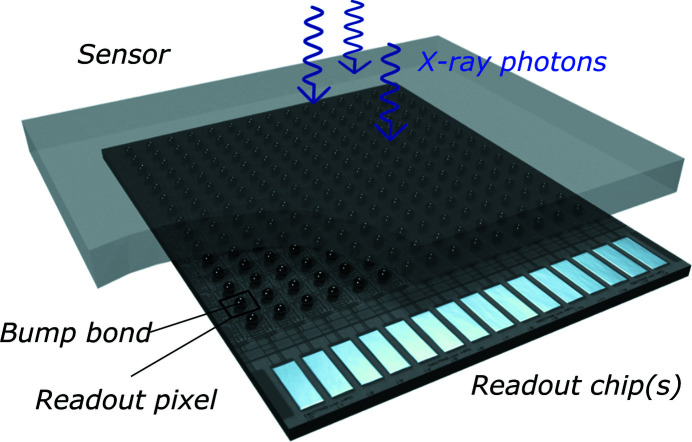
Schematic rendering of an HPC pixel detector.

An HPC pixel detector consists of a pixelated semiconductor sensor, wherein each pixel is connected to its own readout pixel of an underlying readout chip (Fig. 1 ▸). This way, each pixel has photon-counting circuitry and is an independent detector. Photons absorbed by the sensor create a charge cloud that is proportional to the photon energy. With a bias voltage applied across the sensor, the cloud is collected to the input of the readout pixel, creating a charge pulse. When the photon energy exceeds the detector’s adjustable threshold energy, the readout pixel counts the photon. This combination of direct X-ray detection in the sensor and single-photon-counting chip results in favourable detector performance parameters: high quantum efficiency (QE) and optimal spatial resolution described by a one-pixel-wide point spread function (PSF) are enabled by direct detection, while high dynamic range (DR) and frame rate are enabled by the photon-counting ASIC (see definitions of QE, PSF and DR in Appendix *A* [App appa])*.*

The HPC technology’s ‘hybrid’ design allows separate optimization of the readout chip and the sensor, which enables various detector designs. This yielded readout chips with different properties, *e.g.* PILATUS (Brönnimann *et al.*, 2001 ▸), EIGER (Dinapoli *et al.*, 2011 ▸), the Medipix and Timepix family of chips (Campbell, 2011 ▸; Ballabriga *et al.*, 2011 ▸, 2013 ▸, 2018 ▸, 2020 ▸; Wong *et al.*, 2020 ▸; Sriskaran *et al.*, 2020 ▸; Llopart *et al.*, 2022 ▸), PIXIE (Pacella *et al.*, 2008 ▸), PXD18k (Maj *et al.*, 2013 ▸), UFXC32k (Grybos *et al.*, 2016 ▸), IBEX (Bochenek *et al.*, 2018 ▸) and FRIC (Otfinowski *et al.*, 2020 ▸). While the majority of readout chips were developed for use with a silicon sensor, their low absorption efficiency for hard X-rays prompted further developments: sensor materials with high efficiency for energies above 20 keV (*e.g.* CdTe and GaAs), and readout chips that are compatible with these materials. Advanced production of CdTe materials enabled their use even for very large area detectors (Šišak Jung *et al.*, 2017 ▸; Pennicard *et al.*, 2017 ▸).

The evolution of HPC technology continues in parallel with its exploitation at synchrotron beamlines and in laboratories. One of the latest HPC developments is the EIGER2 readout chip (independent of the EIGER chip). Since 2018, the EIGER2 chip was used to build various detectors also called ‘EIGER2’, first with Si sensors, and from 2019 also with CdTe sensors (DECTRIS Ltd, Switzerland). In 2022, a detector firmware upgrade enabled four novel functionalities; the rollout was referred to as a ‘feature upgrade’. At synchrotron sources, these detectors have been used to develop new methodologies or to improve the existing ones. For example, in the field of macromolecular crystallography (MX), this includes serial MX, use of high-energy X-rays for reducing radiation damage (Storm *et al.*, 2021 ▸), and developing a gold standard (Bernstein *et al.*, 2020 ▸). New methodologies were also devised for collecting high-resolution powder X-ray diffraction (PXRD) data (Fitch & Dejoie, 2021 ▸; Dejoie *et al.*, 2018 ▸), as well as for time-resolved measurements: millisecond time scale for small-angle X-ray scattering (SAXS) in solution (Berntsson *et al.*, 2022 ▸) and ultra-small-angle X-ray scattering (USAXS) (Narayanan *et al.*, 2022 ▸), phase-transition monitoring in a diamond anvil cell (Poręba *et al.*, 2023 ▸) and *in situ* Laue diffraction (Zhang *et al.*, 2022 ▸). The combination of modern synchrotron sources and EIGER2 features helped to advance scanning techniques such as ptychography (Kahnt *et al.*, 2021 ▸; Jones *et al.*, 2022 ▸) and biomedical imaging (Giaccaglia, 2022 ▸). EIGER2 detectors were also used in laboratory settings, *e.g.* to facilitate the transition of X-ray absorption spectroscopy (XAS) to laboratory spectrometers (Malzer *et al.*, 2021 ▸; Zimmermann *et al.*, 2020 ▸; Schlesiger *et al.*, 2020 ▸), *operando* SAXS on batteries (Prehal *et al.*, 2022 ▸), nano-computed tomography (Werny *et al.*, 2022 ▸), micro-computed tomography (Solem *et al.*, 2021 ▸; Müller *et al.*, 2021 ▸) and plenoptic X-ray microscopy (Sowa *et al.*, 2020 ▸).

Given the continuous development of EIGER2 detectors and their wide application scope, the aim of this paper is to facilitate their use at synchrotron sources by:

(i) Summarizing the design and performance of the EIGER2 systems including the new functionalities with the feature upgrade (Section 2).

(ii) Explaining the calibrations and corrections relevant for obtaining optimal data quality (Section 3).

(iii) Presenting how EIGER2 can be used to advance data collection strategies in MX, PXRD, ptychography and pump–probe experiments (Section 4).

## 2. EIGER2 detector system and feature upgrade

The EIGER2 series of HPC pixel detectors are based on the same novel readout chip, whose pixels are 75 µm × 75 µm in size and have two energy discriminators. The detectors in the series differ in their sensor type (Si or CdTe for photon energies of about 3.5–40 keV and 8–100 keV, respectively), pixel array size, and calibration for synchrotron or laboratory use. Moreover, the recent feature upgrade of the firmware and software enabled four new functionalities of synchrotron detectors: (i) 8-bit readout mode for doubling the maximum frame rates (Section 2.4.1), (ii) double gating mode (Section 2.5.1) for parallel measurement of two time delays in pump–probe measurements, (iii) streaming of multiple images, *i.e.* of one or both thresholds, or their difference image, at the full detector bandwidth (Section 2.3), and (iv) lines region-of-interest (Lines-ROI) mode for frame rates of up to 98 kHz on a reduced area (Section 2.4.2). This paper focuses on EIGER2 detectors for synchrotron sources, and in Section 4 presents the use of these new functionalities in various applications.

### 2.1. EIGER2 readout chip

**Figure 2:**
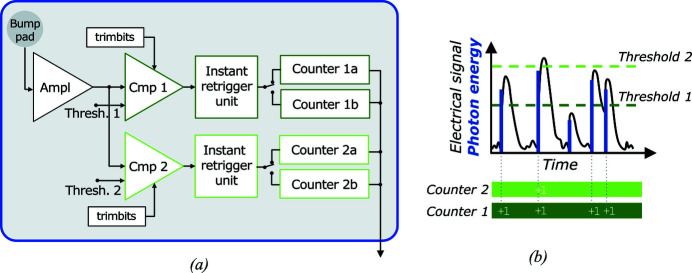
(*a*) EIGER2 readout pixel electronic-circuit scheme whose components are described in the main text. (*b*) Illustration of the principle of photon-counting detection with two thresholds.

The EIGER2 readout chip is an application-specific integrated circuit (ASIC) with an array of 256 × 256 readout pixels [Fig. 2 ▸(*a*)]. Its components can be described following the detection process:

(i) A charge-sensitive amplifier (Ampl) with adjustable gain receives the charge pulse generated in the sensor, and amplifies and shapes the signal.

(ii) The signal enters the two parallel energy-discriminating threshold stages (two comparators, Cmp1 and Cmp2) that operate as low-level discriminators. This permits images for two different energy thresholds to be recorded simultaneously, from the same photon signal. In default operation, Cmp1 sets a lower threshold (*E*_th1_) below the incoming photon energy. Cmp2 can be enabled and used as an upper threshold (*E*_th2_), for detecting signals with energy above *E*_th2_. Both thresholds are independently adjustable (Table 1 ▸). When a signal is higher than *E*_th1_ or *E*_th2_, the corresponding comparator stage generates a digital pulse at its output.

(iii) The instant retrigger unit (Loeliger *et al.*, 2012 ▸) prevents count-rate losses and paralysation of the detector due to a potential pulse pile-up (Brönnimann & Trüb, 2016 ▸). Pile-up refers to pulses that arrive close in time, typically at very high count rates. The resulting pulse is overlapped and wider, and may create a single count rather than multiple counts, causing paralysation of the counting mechanism. Instant retrigger circumvents this by forcing additional count signals whenever the digital pulse exceeds a retrigger time, *t*_r_, which is adjustable and set to be slightly longer than a typical pulse length. This leads to a count rate response that is monotonically increasing with increasing photon flux, and approaching the rate of 1/*t*_r_. The retrigger hence allows for reliable processing of high count rates exceeding 10 Mcounts s^−1^ pixel^−1^. The instant retrigger can be disabled. This is useful for measurement of polychromatic radiation, in which the retrigger could induce an unwanted over-weighting of high photon energies. However, even with retrigger disabled, the maximum count rate still exceeds 5 Mcounts s^−1^ pixel^−1^ (see Fig. 4, Section 2.6).

(iv) The signals coming from the instant retrigger stage cause an increment of the digital counter. EIGER2 has two digital 16-bit counters *for each* of the two energy-discriminating thresholds (Counter 1a/b and Counter 2a/b in Fig. 2 ▸), allowing continuous readout, where one counter (a or b) is counting the photons for the current image while the counts of the previous image are read from the other counter. Switching between the two counters takes 100 ns, *i.e.* a negligible dead-time between two exposures, and guarantees a duty cycle of above 99%, even at the maximum 98 kHz frame rate possible (Lines-ROI, Section 2.4.2). The 16-bit counters can be operated in an 8-bit mode, reducing the amount of data that need to be read out, hence allowing to double the readout speed. The two counters of a threshold could also be concatenated to form a single 32-bit counter. However, this is normally avoided to keep the advantage of the continuous readout. Instead, 32-bit images are generated by auto-summation (Section 2.4.1).

Electronic gating of the counting process is possible with gating times below 60 ns. The two counters (a and b) can be independently gated, enabling a new *double-gating* mode (Section 2.5.1).

The EIGER2 ASIC design is compatible with silicon and CdTe sensors, for which it is operated in hole and electron collection mode, respectively. For this, pixel inputs of the ASIC can accept charge signals of either polarity, with a high voltage (HV) of corresponding sign being applied to the sensor contact on the X-ray entrance side (HV > 0 for Si and HV < 0 for CdTe).

### 2.2. EIGER2 modules and multi-module systems

EIGER2 detectors are built from detector modules with an active area of 77.1 mm × 38.4 mm (1028 × 512 pixels), each module comprising 4 × 2 readout chips. The readout chips are three-side buttable and connected on the fourth side *via* the wire bond pads (Fig. 1 ▸). A single Si sensor covers the entire module. For CdTe detectors, two CdTe sensors are used to cover 2 × 2 readout ASICs of the module, with a gap of two pixels. The sensors are active over their entire surface, and bridge the gap of two pixels between ASICs by sensor pixels of double size.

Large detectors can be built as multi-module arrays. The largest EIGER2 detectors have an array of 4 × 8 modules, with about 16 million pixels. The modules are mounted on a water-cooled base plate for temperature stabilization at around room temperature. The active cooling also permits vacuum-compatible EIGER2 detectors to be built, which reach vacuum pressures below 10^−3^ mbar, or even down to 10^−6^ mbar when placing readout electronics outside the vacuum chamber.

### 2.3. Sensor materials and quantum efficiency: Si and CdTe

High-quality silicon (Si) sensors are available thanks to the high degree of maturity of the silicon technology from the semiconductor industry. They feature excellent quantum efficiency in the range of about 5–15 keV, but above 25 keV their absorption efficiency falls off and sensor material of higher atomic number has to be used. Definitions are given in Appendix *A* [App appa].

The preferred material is cadmium telluride (CdTe), because of its high efficiency to above 50 keV and its availability as a detector-grade material. However, CdTe wafers still show some limitations in size and quality (Pennicard *et al.*, 2017 ▸). In particular, remaining lattice defects and impurities can trap the charge and cause polarization. This non-permanent effect becomes more relevant at high photon energies, high fluxes and long exposure times. It is mitigated by optimal sensor bias voltage and temperature operating conditions.

**Figure 3:**
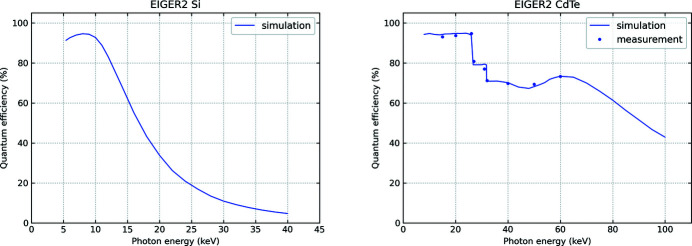
Quantum efficiency of EIGER2 as a function of photon energy for 0.45 mm-thick Si (left) and 0.75 mm-thick CdTe (right) detectors with threshold set to 50% of the photon energy.

EIGER2 has 0.45 mm-thick silicon sensors or 0.75 mm-thick CdTe sensors, and the corresponding QE is presented in Fig. 3 ▸ (QE is defined in Appendix *A* [App appa].)

The QE was determined using a Monte Carlo simulation code (Trueb *et al.*, 2017 ▸) that includes many effects of the detection process. The QE plot of CdTe shows a drop at 26.7 keV and at 31.8 keV (*K*-edges of Cd and Te, respectively), which is due to events in which a fluorescence photon is re-emitted, and leaves the sensor, or is absorbed in a neighbouring pixel, whereby the charge deposited by the event in either pixel becomes too small to exceed the detector’s energy threshold.

The experimental QE of an EIGER2 CdTe module was determined at the BAMline (Görner *et al.*, 2001 ▸) at BESSY II, using silicon photodiodes calibrated against a cryogenic radiometer as primary standard in the hard X-ray range (Gerlach *et al.*, 2008 ▸). A 750 µm-thick CdTe sensor was measured for several photon energies, with the energy threshold set to half of the photon energy. The experimental data (Fig. 3 ▸, right) match the simulated curve within an estimated error of the QE measurement of 1–2%.

### 2.4. Image acquisition and readout

The acquisition and readout of images is controlled *via* the detector control unit (DCU), which connects to the detector and provides the user interface.

Control and configuration parameters are passed to the DCU using an HTTP-based REST-like application programmer interface (API) (SIMPLON 1.8 API, Version 2.1, DECTRIS, Switzerland).

Image readout from the DCU is possible over an Ethernet connection by using one of the interfaces: (i) the stream interface for fast data transfer at the maximum bandwidth, allowing for on-line visualization and/or processing, or, writing the data to a file system; (ii) the filewriter interface using the HDF5 container file format and creating data sets of hundreds to thousands of images with NEXUS style headers (Bernstein *et al.*, 2020 ▸) retrievable by the user from the DCU *via* the Ethernet interface; and (iii) a monitor interface transferring TIFF images into an image buffer for low-frame-rate applications. The acquired HDF5 or TIFF files can be opened and viewed with the image viewer *ALBULA* (Pilipp, 2014 ▸) and many other applications supporting runtime decompression of LZ4/BSLZ4 data.

The detector may be used with only one energy threshold enabled, or both thresholds can be activated to obtain images with different spectral information. Moreover, it is possible to automatically subtract the signal from the upper threshold from the lower threshold and deliver the difference image, similar to an energy window. For example, by setting the upper threshold to above the photon energy, high energetic background such as natural radiation background or higher harmonic radiation can be suppressed in the difference image (see Appendix *B* [App appb]). As part of the features upgrade, the stream readout interface was upgraded to ‘STREAM2’, which enables multiple images per exposure to be received continuously from the detector, using the detector’s full data bandwidth. The detector output can be configured to deliver a single threshold image, or either combination of upper- and lower-threshold images, and the difference image. Application examples are given in Sections 4.2.1 and 4.2.2.

#### 2.4.1. Readout bit depth and auto-summation

The internally used counter bit depth can be different from the image bit depth. For a given exposure time and frame rate, the required image bit depth for the output is automatically selected: 16-, 32- or 8-bits. By default, the 16-bit counters of a threshold are read out alternately at their full 16-bit depth to allow for a continuous readout with high duty cycle. To achieve a higher dynamic range of 32-bit for the output image, the detector’s ‘auto-summation’ functionality automatically splits the acquisition into multiple exposures and integrates many 16-bit readouts into a 32-bit image, internally on the DCU. This auto-summation process extends the dynamic range from 16-bit (maximum 65535 counts) up to 32-bit (maximum 4.3 × 10^9^ counts), without loss of data quality (see Appendix A2). Auto-summation is performed only up to a certain frame rate. At high frame rates, where the exposure time is not long enough for a pixel to reach the counter limit at the maximum count rate, the detector will provide 16-bit data. For even higher frame rates, which would exceed the data bandwidth limit, the detector switches into the 8-bit mode, reading only eight bits from each 16-bit counter.

#### 2.4.2. Data bandwidth and maximum frame rates

Between the detector and the DCU, the detector readout board with four 10 Gigabit Ethernet (GbE) connections sets the bandwidth limit of 40 Gb s^−1^ for data transfer. This bandwidth limit determines the maximum frame rate for any given detector size (number of modules) and image bit depth. The resulting maximum frame rate reaches up to 2250 Hz in 16-bit mode, and up to 4500 Hz in 8-bit mode, for readout of a single threshold from a detector with up to two modules (∼1 million pixels) and a single readout board. For larger detectors, the maximum frame rate scales with the number of readout boards and inversely with the number of modules. EIGER2 XE systems with four readout boards yielding a total data bandwidth of 160 Gb s^−1^ increase the maximum frame rate up to four times.

For large multi-modular EIGER2 systems, higher frame rates can be achieved by reading a region of interest (ROI) comprising a fixed sub-set of modules of the detector array. Depending on the detector’s readout bandwidth, this allows the ROI to be read at an increased frame rate, thereby emulating a smaller detector with higher frame rate.

As a part of the feature upgrade, Lines-ROI readout mode was introduced to increase the detector’s frame rate by reducing the readout area to a symmetric region around the vertical centre of the detector modules. The number of lines (pixel rows) which defines the height of the ROI can be selected by the user up to the full 512 lines of the module. The reduction of ROI height increases the maximum frame rate proportionally, up to 98 kHz, which allows for optimization of active area versus speed specifically for each experiment. For example, for a single or two-module wide detector, an ROI with a height of 96 lines, in 8-bit readout mode, allows for a frame rate of up to 24.5 kHz compared with 4500 Hz for the full-frame readout. An application example is given in Section 4.3.

#### 2.4.3. Image compression

Image compression on the fly is possible using the lossless LZ4 or BSLZ4 (Github-lz4, https://github.com/lz4/lz4; Github-bitshuffle, https://github.com/kiyo-masui/bitshuffle) compression algorithms to reduce the size of the recorded images and the required data bandwidth for saving the data to a network. For macromolecular crystallography data, compression ratios between 6.5 and 14.4 are reached depending on the acquisition strategy, with strategies with sparser diffraction images, containing fewer photon counts per image, leading to higher compression ratios (Förster *et al.*, 2016 ▸). For fast-scanning ptychography data (application example in Section 4.4) where there are many zero-valued pixels, BSLZ4 compression ratios between 100 and 200 are common.

### 2.5. Trigger and gating

Several gating and trigger modes are available on EIGER2 detectors, to synchronize the acquisition to the experiment with a TTL-type signal provided to the detector. Trigger signal rising edges start the acquisition of an image, or a series of multiple images. The gating mechanism, also called an ‘electronic shutter’, directly controls the pixel circuitry and activates the pixels for counting for the precise time during which the provided gate signal is at HIGH level, with time resolution down to below 60 ns. The gating mechanism allows for a single gate signal, or a series of gate signals, to be sent for exposing multiple times prior to image readout, which is especially useful in stroboscopic measurements.

#### 2.5.1. Double-gating mode

The double-gating mode introduced with the feature upgrade makes use of the two separately gateable pixel counters, a and b [*cf*. Fig. 2 ▸(*a*)]. By alternately activating counters a and b for an incoming consecutive sequence of gate signals, two separate images can be acquired in counters a and b. The first and all following odd-numbered gate signals activate counter a, while the second and all even-numbered gate signals activate counter b. Images of the state before and after the sample pump or reaction start can thus be collected within the same exposure time, enabling perfect background correction (see application example in Section 4.5).

### 2.6. Count-rate characteristics

The count-rate curve of an HPC detector describes the measured count rate as a function of the incoming rate of photon events. It depends on parameters which influence the length of digital pulses in the readout circuit: photon energy, corresponding amplifier gain used by the detector at this energy, and energy threshold.

**Figure 4:**
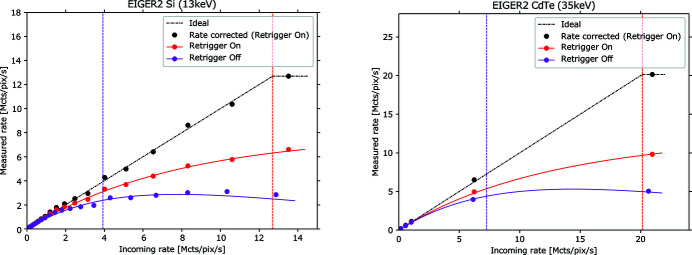
Comparison of acquisition with instant retrigger disabled (‘Retrigger Off’, purple) and enabled (‘Retrigger On’, red) for EIGER2 detectors with Si (left) and CdTe (right) sensors. Count-rate curves used for the count-rate correction (solid lines) are plotted with the result of verification measurements (circles). The count-rate correction for the data measured with ‘Retrigger On’ gets very close to the linear (‘Ideal’). The vertical dashed lines indicate the ‘cutoff’ count rate up to which EIGER2 performs count-rate correction at these photon energies (*cf*. Section 3.2). The ‘cutoff’ with retrigger mode disabled (purple dashed lines) lies at about 4 Mcounts s^−1^ pixel^−1^ for Si and 7.2 Mcounts s^−1^ pixel^−1^ for CdTe detectors, and with the retrigger mode enabled (red dashed line) at about 12.5 Mcounts s^−1^ pixel^−1^ for Si and 20 Mcounts s^−1^ pixel^−1^ for CdTe detectors.

Fig. 4 ▸ shows count-rate curves of EIGER2 detectors with Si and CdTe sensors, for typical photon energies for Si (13 keV) and CdTe (35 keV), respectively. Without the instant retrigger enabled (‘Retrigger Off’), the count-rate curves reach a maximum ‘saturation point’ at an incoming rate of ∼7 Mcounts s^−1^ pixel^−1^ and ∼14 Mcounts s^−1^ pixel^−1^ for Si and CdTe, respectively, after which they fall off. Using the retrigger mode (‘Retrigger On’) for non-paralyzable counting, the measured count-rate curves become steeper and monotonically increasing. This enables measurements at higher incoming rates and removes the ambiguity whether the measured rate was created by an incoming photon rate below or above the saturation point.

By default, EIGER2 detectors apply the appropriate count-rate curve for the count-rate correction of measured signals up to a certain ‘cutoff’ value in Fig. 4 ▸. Further details are given in Section 3.2. Higher photon energies allow lower preamplifier gain settings, resulting in improved count-rate performance. With retrigger enabled and the threshold set to 50% of the photon energy, EIGER2 detectors achieve a maximum (‘cutoff’) count-rate ability of at least 3.8 Mcounts s^−1^ pixel^−1^ for Si (<10 keV) and at least 8.3 Mcounts s^−1^ pixel^−1^ for CdTe (<11 keV), while performing better than 10 Mcounts s^−1^ pixel^−1^ above 12.4 keV, for both Si and CdTe.

## 3. EIGER2 calibration and corrections

To achieve an optimal measurement result with equal response of all pixels over the entire count-rate range, calibration and correction data are acquired and stored for each detector. Calibration data equalize the energy thresholds prior to measurements. Moreover, corrections to the pixel values of the acquired images are applied on-the-fly during the measurement based on the calibration data on the detector control unit.

### 3.1. Calibration of the energy thresholds

In the calibration of comparator voltage (thresholds) to photon energy, variations between individual comparator stages are equalized by fine-tuning the value of the threshold with a dedicated trimming circuitry, one for each comparator [Fig. 2 ▸(*a*)]. In this procedure (Kraft *et al.*, 2009 ▸; Zambon *et al.*, 2019 ▸), detectors are illuminated with radiation from fluorescence targets, and the position of fluorescence *K*α lines at different photon energies is determined from scans of the comparator voltage for several fluorescence targets. The energy calibration is stable and only needs to be performed once. However, it is known to slightly shift with change of temperature, hence EIGER2 detectors are stabilized with a cooling water circuit that is close to room temperature.

### 3.2. Corrections

In order to deliver the optimal data quality, the EIGER2 detector by default performs several data corrections. While it is generally recommended to keep these corrections enabled, for specific experiments they can be individually disabled.

#### 3.2.1. Count-rate correction

An ideal photon counter would feature a linear response with count rate. The deviation of a real detector such as EIGER2 from the ideal counter (Fig. 4 ▸) can be taken into account by the built-in count-rate (linearity) correction. This count-rate correction converts the counts measured in a pixel into the corresponding number of incident photons that would have been recorded by the ideal photon counter. This correction is based on the inversion of the count-rate curve, wherein the measured count rate in a pixel is calculated as the ratio of measured counts over the exposure time. The count-rate correction is performed up to a boundary value (‘cutoff’), up to which the curves can be reliably rectified (inverted). The cutoff is set as the count rate at which the slope of the count-rate curve falls below a certain value, below a slope of 0.2 for the default setting ‘Retrigger On’, and below a slope of 0.3 for ‘Retrigger Off’. Pixel values corresponding to higher count rates will be masked (set to the maximum corrected count at the cutoff rate). When recording images with the auto-summation functionality, rate correction is performed for each individual sub-frame before summing. This is important to assure the most accurate measurement for fast varying signal intensities, *e.g.* scanning over a Bragg peak in single-crystal diffraction.

#### 3.2.2. Flatfield correction

The in-built flatfield correction, sometimes referred to as gain correction, equalizes pixel-to-pixel sensitivity variations. The correction is based on flatfields, *i.e.* detector response to homogeneous illumination of defined X-ray energy. The factory calibration relies on illumination by several fluorescence targets with a different emission energy. Flatfields are recorded for each target (X-ray energy) at two different energy thresholds: one corresponding to 50% (default setting) and the other to 70% of the photon energy. For EIGER2, flatfields are recorded with an average of about 100000 counts per pixel. This achieves high data quality because the signal-to-noise ratio (SNR) of the pixel value in a corrected image can only be as good as the one in the flatfield image. The variation of measured counts in each pixel follows statistics close to a Poisson distribution. Hence, for 100000 counts in the flatfield, it has SNR_flat_ = (1/100000)^1/2^ = 1/316, *i.e.* the SNR_flat_ enables accuracy down to 0.3% in each pixel. Integration over several pixels will enhance the achievable data quality even further.

In EIGER2 detectors, photon energy and threshold can be continuously set within the defined range (Table 1 ▸). For settings of the energy and/or threshold between the calibration points, the DCU calculates an interpolated flatfield from the factory flatfields by linear 2D interpolation.

#### 3.2.3. Bad pixels and pixel masking

For EIGER2 systems, the quality-controlled number of ‘bad’ pixels is <0.05% on Si and <0.1% on CdTe detectors. Bad pixels are classified into different types: dead, under- or over-responding, or noisy. A ‘pixel mask’ is a coded image containing non-zero values for each bad pixel, with the value indicating its classification; for multi-module detectors, pixels in the ‘gap’ are also masked. The pixel masking on EIGER2 replaces the value of bad pixels in recorded images with the maximum value of the current image output format (*e.g.* 2^8^ − 1 = 255 for 8-bit output), or alternatively with zeros, which achieves higher compression ratios and can be handy for the quick presentation of image data to avoid auto-scaling issues. It is possible to disable the pixel masking.

Gaps between neighbouring readout chips are bridged by two rows or columns of larger sensor pixels (75 µm × 150 µm). This maintains the pitch of the normal-sized pixels in the images while keeping a fully active sensor area. Counts registered by these larger pixels are redistributed by software into two normal-sized image pixels, of which the one lying on the ‘gap’ is refered to as a ‘virtual pixel’, using the detector’s virtual-pixel correction according to the principle described by Kraft *et al.* (2009 ▸). Alternatively, the virtual pixel correction can be switched off leaving this area masked.

#### 3.2.4. Experiment-specific corrections

Factory flatfields are limited to X-ray energies available in a laboratory (spectra from fluorescence targets). While the provided flatfields work well for a wide range of X-ray energies, in some cases it is advisable to collect experiment-specific flatfield data, for the exact parameters that will be used in a subsequent measurement. These cases include:

(i) Using energy thresholds that significantly deviate from the calibrated settings for a given X-ray energy and threshold.

(ii) Significant discrepancy between X-ray energy of the experiment and the energy at which the flatfield was taken (including polychromatic irradiation).

When many pixels with low signal intensity are summed or averaged, *e.g.* in azimuthal integration of low-intensity PXRD or SAXS/WAXS patterns, it can be beneficial to deactivate the flatfield correction for the measurement. This circumvents rounding errors on low-intensity signals due to the representation of the corrected detector images as integer values. In such a case, the appropriate flatfield can be retrieved from the detector metadata during post-processing to implement the flatfield correction as part of the data processing routines.

Other experiment-specific corrections include parallax correction, efficiency correction and geometry correction for module offsets, which are implemented and can be refined in some application-specific data-processing software for single-crystal diffraction, *e.g.* *XDS* (Kabsch, 2010 ▸) and *DIALS* (Winter *et al.*, 2018 ▸). The geometry correction relates to the mechanical positioning offset of sensors, *i.e.* the position of the pixel grid on a sensor relative to the overall grid of the output image, which is of the order of half a pixel (75/2 µm) for EIGER2 detectors. Wright *et al.* (2022 ▸) acquired PXRD patterns to determine the module offsets, and, by correction for the measured module offsets, demonstrated the ability to achieve a position accuracy of 1/350 pixel.

## 4. X-ray applications

EIGER2 detectors have been used for X-ray diffraction, scattering, spectroscopy and imaging applications. The following subsections highlight examples of how EIGER2 enables new data collection strategies and achieves faster data collection and cleaner data at synchrotron sources. The examples of Sections 4.2 [Sec sec4.3] to 4.4 focus on the novel detector functionalities.

### 4.1. Macromolecular crystallography

Since their introduction in 2006, HPC detectors have fundamentally transformed macromolecular crystallography (Förster & Schulze-Briese, 2019 ▸). In the meantime, the leading role of crystallography for the determination of the three-dimensional (3D) structure of macromolecules was challenged by single-particle analysis cryo-electron microscopy (see *The Nobel Prize in Chemistry*, https://www.nobelprize.org/prizes/chemistry/2017/summary/), as well as artificial-intelligence-based structure prediction methods (Method of the Year 2021, 2022 ▸). Nowadays, the combination of EIGER2 detectors, advanced beamline instrumentation and intelligent data acquisition workflows offers the potential to overcome the limitations of the aforementioned methods in terms of throughput and accuracy of the structural information.

Allowing for elucidating of the 3D molecular structure of proteins, nucleic acids and their complexes with small molecules (Burley, 2021 ▸), structural biology has an ever-growing impact on drug development up to the approval of new drugs by the Food and Drug Administration (Westbrook & Burley, 2019 ▸). High throughput is a prerequisite to efficient structure-based drug discovery (SBDD), including screening of drug libraries (Günther *et al.*, 2021 ▸) as well as fragment-based lead discovery (Schiebel *et al.*, 2016 ▸). The combination of advanced synchrotron beamline components and highly stable automation software has enabled data collection times as short as 15 s at beamline I04-1 at Diamond Light Source, UK. The upgrade of the beamline with an EIGER2 XE 9M detector and a hybrid permanent-magnet undulator (HPMU) resulted in a five-fold increase of the throughput, equivalent to more than 225000 crystals per year (Diamond Light Source, 2020 ▸).

Another example of high throughput for SBDD is an optimized workflow for collecting highly accurate data by minimizing systematic errors at beamline P14 of the PETRA III synchrotron. This is achieved by combining the EIGER2 X CdTe 16M detector, a flat-topped beam profile at 26.7 keV, a multi-axis goniostat, and the workflow developed by Global Phasing Ltd. This workflow follows several steps: (i) determining the crystal symmetry and orientation, (ii) designing a multi-orientation strategy to achieve completeness (no cusps) and maximize uniformity of redundancy, within a ‘dose budget’ adapted to the target resolution, and (iii) driving the execution of that strategy *via* the beamline control software *MXCuBE2* (Oscarsson *et al.*, 2019 ▸). In addition to the high quantum efficiency, EIGER2 CdTe detectors offer a parallax reduction by more than an order of magnitude relative to Si sensors, thanks to the short absorption length of CdTe at high energies. In the workflow the reduced parallax allows for two distinct advantages: (*a*) it increases the signal-to-noise ratio of high-resolution reflections improving the diffraction limit of the data, and (*b*) it facilitates the integration of densely spaced reflections.

**Figure 5:**
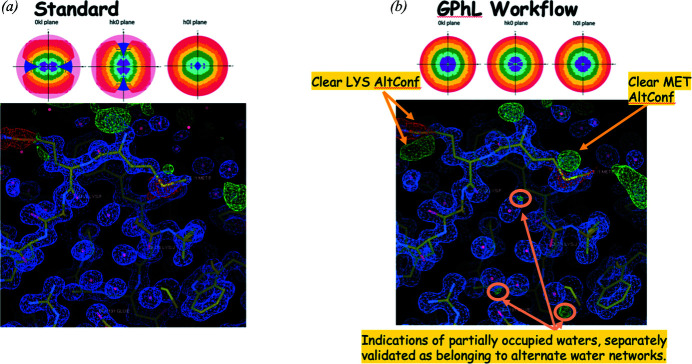
Electron density maps resulting from (*a*) standard data acquisition protocol and (*b*) Global Phasing workflow. The latter shows evidence of alternate conformations of specific residues as well as partially occupied waters. The top panel displays STARANISO plots of 〈*I*/σ(*I*)〉 (STARANISO anisotropy and Bayesian estimation server, https://staraniso.globalphasing.org/cgi-bin/staraniso.cgi), demonstrating the homogeneous completeness achieved with the Global Phasing workflow.

This setup has resulted in several spectacular datasets. For example, for a primitive orthorhombic system with 560 kDa per asymmetric unit, it yielded a 0.98 Å resolution dataset with almost 100M reflections, 2.75M unique, using a total dose of 2.5 MGy (*cf.* Fig. 5 ▸ and Appendix *C* [App appc]) (Chari *et al.*, 2023 ▸).

The optimal photon energy in terms of diffraction efficiency, defined as the number of elastically scattered photons per unit dose, has been the subject of discussion in the crystallographic community for decades (Arndt, 1984 ▸; Fourme *et al.*, 2012 ▸). Theoretical diffraction-physics-based studies predict an optimum at approximately 35 keV (Arndt, 1984 ▸; Dickerson & Garman, 2019 ▸). While most former detectors had poor QE at high energy, the recent introduction of CdTe sensor material allows the potential of high-energy data-collection to be exploited. According to simulations by Dickerson & Garman (2019 ▸), data collection just below the *K*-edge of Cd (26.7 keV) maximizes the diffraction efficiency. For the combination of beams smaller than 5 µm and high-resolution shells, a gain of up to four can be reached at this energy as compared with data collected at 12.4 keV. Storm *et al.* (2021 ▸) confirmed that the factor is greater than two for beam sizes of the order of 9 µm, and demonstrated a statistically significant increase of the mean resolution cutoff between 12.4 and 25 keV. The team also verified the data quality of the EIGER2 X CdTe 9M detector at 25 keV to be superior to data collected at 12.4 keV with a PILATUS3 X 6M, as judged by standard data quality indicators [*R*_meas_, 〈*I*/σ(*I*)〉].

### 4.2. Powder X-ray diffraction

In the last decade, two-dimensional PXRD (2D-PXRD) has become a common data collection strategy. This section demonstrates how the novel features of the EIGER2 detectors at synchrotron sources are used to suppress noise, background radiation and higher harmonics of undulator radiation, improve peak shapes, and increase data collection speed.

#### 4.2.1. High-resolution powder X-ray diffraction

The high-resolution powder X-ray diffraction beamline at the ESRF (ID22) is optimized for high-flux high-angular-resolution experiments in the energy range 6–80 keV. Prior to the ESRF upgrade (in 2019), the beamline employed a nine-channel Si 111 multi-analyser stage with scintillation detectors, scanned over the desired 2θ range (Hodeau *et al.*, 1998 ▸), which allowed for accurate determination of 2θ angles and a very narrow instrumental profile [full width at half-maximum (FWHM) (LaB_6_) < 0.0025° 2θ].

Although this system operated successfully for over 20 years, some drawbacks remained. In particular, the width of the 4 mm-wide fixed axial receiving slit had been chosen as a compromise between low-angle resolution and peak shape versus high-angle counting efficiency. Indeed, at low diffraction angle, the axial acceptance needs to be quite narrow to avoid broadened and asymmetric peaks arising from the curvature of the Debye–Scherrer cones. At high diffraction angles, where asymmetry is no longer such a problem, detection efficiency could be improved by increasing the axial acceptance as the scattering power of the sample falls off naturally. The provision of new detector technology allows these issues to be addressed without compromise.

**Figure 6:**
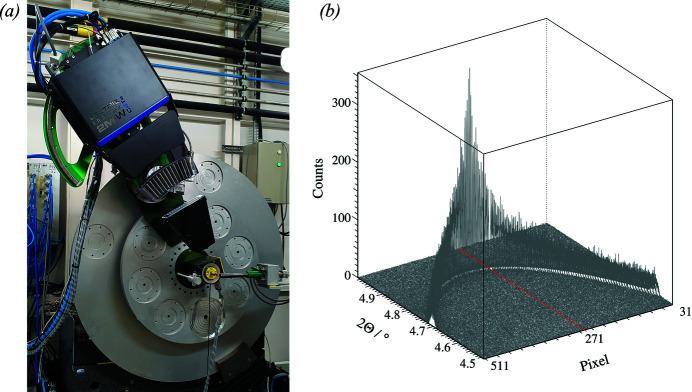
(*a*) Two-circle diffractometer and 13-crystal multi-analyser stage with EIGER2 2M-W detector at the ID22 beamline. (*b*) LaB_6_ 100 reflection (λ = 0.3542 Å) as a function of the detector pixel columns 31–511. The further from the centerline of the detector, the lower the apparent 2θ angle, and the broader the diffraction peak. The axial resolution provided by the EIGER2 detector allows the 2θ scale to be corrected along with mitigation of the broadening effect.

After the ESRF upgrade in 2019, the ID22 beamline has replaced the previous high-resolution setup with a 13-crystal analyser stage and an EIGER2 X CdTe 2M-W pixel detector [Fig. 6 ▸(*a*)]. Diffraction data are collected while continuously scanning the detector arm at speeds from 1 to 30° per minute, with corresponding readout frequencies of the detector from 33 to 1000 Hz. Higher harmonics of the undulator and cosmic rays are rejected by using the detector in difference mode. Diffraction signals from the sample are transmitted by the analyser crystals onto 13 regions of interest on the detector (Dejoie *et al.*, 2018 ▸). By exploiting the short dimension of the active area (512 × 4148 pixels) for the axial resolution, the effect of axial divergence, which causes the asymmetry in the peak shape at low 2θ angles of PXRD patterns, can be removed. Asymmetry occurs because axially diverging photons satisfy the Bragg condition at the analyser crystal at lower angles of the detector arm than those scattered closer to the diffraction plane and so appear to be diffracted at lower 2θ angles. By recording the axial position at which a photon arrives at the detector, its true 2θ angle from the sample can be calculated (Fitch & Dejoie, 2021 ▸) [Fig. 6 ▸(*b*)]. The overall effect is that peaks at low angle are more symmetric and narrower when such corrections are applied. A second advantage is that the axial acceptance of the detector can be increased as 2θ increases, up to the 38 mm width of the detector, thus increasing the statistical quality of the high-angle data while also improving the angular resolution. ID22 has implemented this approach systematically, as an automatic procedure, into its collection of high-resolution PXRD data (Fitch *et al.*, 2023 ▸).

#### 4.2.2. Higher-harmonic suppression in PXRD

Beamline ID15B of the ESRF is dedicated to diffraction experiments on samples at pressures generated by a diamond anvil cell (Poręba *et al.*, 2023 ▸). Its working energy is 30 keV, produced by a U20 in-vacuum undulator and a nitro­gen-cooled single-bounce Si (111) monochromator. Normally the beam is focused by two transfocators equipped with one-dimensional compound refractive lenses (CRLs) made from Be. Such a setup does not reject higher harmonics, especially at 90 keV, the ninth harmonic of the undulator passing the (333)-reflection of the monochromator, which creates unwanted artefacts in X-ray diffraction patterns.

**Figure 7:**
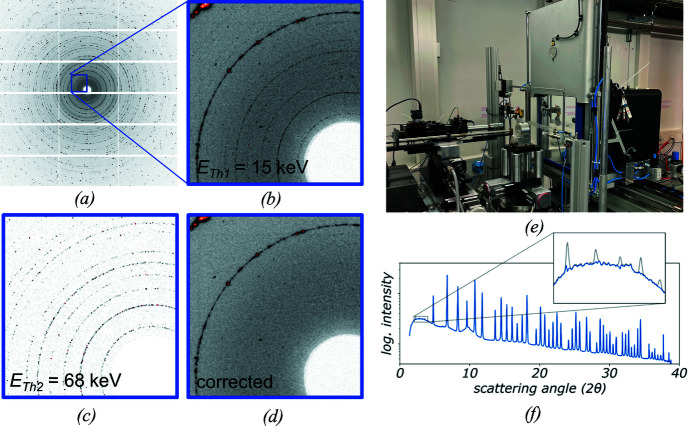
(*a*–*c*) LaB_6_ powder patterns recorded using two energy thresholds; (*e*) EIGER2 X CdTe 9M detector at beamline ESRF ID15B; clear suppression of higher harmonic radiation in (*d*) the corrected image and (*f*) integrated powder pattern.

The possibility of suppressing the higher harmonics was tested using an EIGER2 X 9M CdTe detector. For the experiment, the 30 keV beam was focused by 58 two-dimensional CRLs made from Al, which enhances the relative contribution of higher harmonics at 90 keV even more. PXRD images acquired on an LaB_6_ reference sample [Fig. 7 ▸(*a*)] showed a noticeable contribution of the 90 keV higher-harmonic energy in the form of multiple diffraction rings in the low-angle regime [Figs. 7 ▸(*a*) and 7(*b*), and the grey data line in Fig. 7(*f*)]. In the same measurement, an upper threshold was set to 68 keV to detect the 90 keV contribution, while the lower threshold was kept at the default value of 15 keV, *i.e.* 50% of the photon energy. The images for two thresholds were recorded simultaneously *via* the stream interface, making it possible to isolate the 90 keV scattering contribution [Fig. 1 ▸(*c*)] with the upper threshold and remove it from the fundamental image.

To remove the signal of 90 keV radiation from the lower-threshold image, a correction factor was applied to the upper-threshold image to correct for the significantly different relative threshold-energy to photon-energy ratio for the 90 keV radiation in both images (15/90 = 16.7% versus 68/90 = 75.6%) before subtraction from the lower-threshold image. The corrected image provides clean diffraction data [‘corrected’ image in Fig. 7 ▸(*d*) and the blue powder data line in Fig. 7 ▸(*f*)], with an increased SNR that is achieved without making any further experimental adjustments.

Rejection of higher harmonics with the upper threshold may become of practical use at ID15B, because reflections of the higher harmonics from the large diamond anvils can be mistaken for a signal from the tiny sample inside the cell. Harmonic rejection based on the two threshold images can enable the rejection where suppression by the X-ray optics is not possible. In combination with the STREAM2 interface, this can be even done at high speed using the full detector bandwidth, in time-resolved or scanning applications.

### 4.3. Time-resolved PXRD at 9 kHz

The brilliance of low-emittance fourth-generation synchrotron radiation facilities is several orders of magnitude higher than that of the current ones, offering new opportunities for ultrafast time-resolved research. The Lines-ROI feature of EIGER2 allows acquisitions up to 98 kHz by reducing the readout area to a selectable number of central pixel lines. This brings unprecedented performance and flexibility to time-resolved scattering experiments, especially for the characterization of irreversible processes such as additive manufacturing.

**Figure 8:**
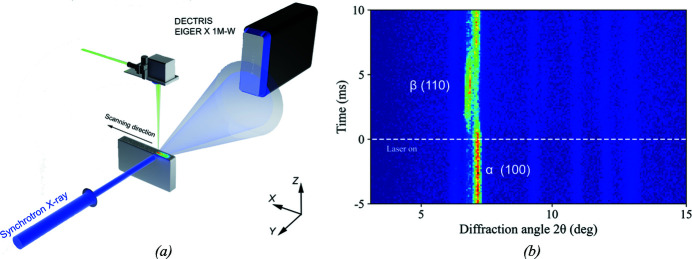
(*a*) Schematic rendering of the *in situ* additive manufacturing setup implemented at BL3W1 of Beijing Synchrotron Radiation Facility. (*b*) The 9 kHz time-resolved diffraction patterns of Ti alloy during a laser melting of 1 ms.

Here, an EIGER2 X CdTe 1M-W detector was positioned at BL3W1 of the Beijing Synchrotron Radiation Facility (People’s Republic of China) to capture the fast melting and solidification dynamics of Ti alloys during additive manufacturing [Fig. 8 ▸(*a*)]. The detector offers a quantum efficiency of about 68% at the employed photon energy of 51.2 keV. In particular, the Lines-ROI feature of the detector was adjusted to read out 2068 × 256 pixels in 8-bit mode, with an effective time resolution of approximately 110 µs (8-bit mode) at a frame rate of 9 kHz. This unambiguously resolved the fast phase transportations (α → β → α) during the additive manufacturing process.

Fig. 8 ▸(*b*) shows the time sequences of diffraction patterns of the sample during the laser melting process, where τ = 0 ms refers to the moment when the laser is on. The high intensity of the α (100) Bragg peak before laser melting indicates an α-phase dominated structure at the static state. After laser irradiation of 1 ms, the intensity of the α (100) peak abruptly drops within the first 1 ms and reappears at approximately τ = 3 ms, accompanied by an angular shift firstly towards the lower angle and then backwards, due to the thermal expansion and the following cooling process. Meanwhile, the β (110) peak emerges and reaches its saturation at τ = 3 ms and then fades away in the following several milliseconds. At τ = 8 ms, the diffraction peak almost returns to its initial state, but still shows a slight peak shift to the lower angle, indicating a residual lattice expansion. Higher frame rates, *e.g.* >20 kHz, could not be achieved due to the poor X-ray flux; however, the current proof-of-principle experiment proves that the EIGER2 detector will have potential in the coming fourth-generation synchrotron facilities, especially in time-resolved experiments.

### 4.4. Ptychography at high scanning speed

Ptychography is a diffractive imaging method that is, like coherent diffractive imaging, used to phase coherent diffraction patterns of aperiodic samples (Miao *et al.*, 1999 ▸; Pfeiffer, 2018 ▸). The technique relies on accurate measurement of the diffraction signals that strongly vary in intensity over the coherent diffraction pattern, but also on optimized scanning speed.

A common feature of most X-ray ptychography experiments is that redundancy is generated in the data by scanning the sample in small steps across a stationary beam. The scan time includes non-measurement overheads: time to accelerate, move, decelerate and settle the sample between scan points, and the time to read out the detector data, which is done in parallel with moving the sample. For large datasets, *e.g.* tomographic scans, this resulted in very long scans and overhead (Dierolf *et al.*, 2010 ▸). Flyscans, wherein the sample continuously moves during acquisitions, were proposed to overcome the stop-motion overheads (Clark *et al.*, 2014 ▸; Pelz *et al.*, 2014 ▸; Huang *et al.*, 2015 ▸; Deng *et al.*, 2015 ▸). However, it was also pointed out that the overlap should be much greater in the scan direction than in the non-scanning direction (Pelz *et al.*, 2014 ▸). In principle, by increasing the detector frame rate, an increase in overlap can be achieved for an equivalent scan time. This amounts to distributing the diffraction intensity over a larger number of frames, where each frame has lower signal-to-noise ratio, but there is inherently more redundancy in the data. For detectors with an appreciable readout time, this possibly requires slowing down a scan, to ensure that the sample does not move too far relative to the beam size between the end of one exposure and the start of the next. But, in the high frame rate limit, the detector readout overheads can become substantial, resulting in an appreciable fraction of the scan time being wasted.

**Figure 9:**
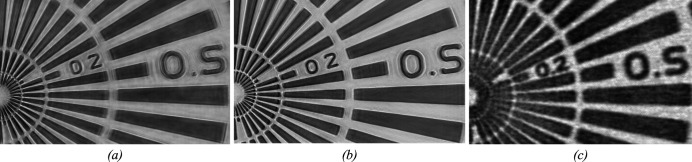
Flyscan ptychography images of a test pattern at 10 keV, (*a*) with the detector triggered at each scan point, spatial resolution 82 nm; (*b*) free-run software-triggered dead-time-free acquisition at 50 Hz frame rate, spatial resolution 70 nm, data collection rate 0.49 µm^2^ s^−1^; (*c*) free-run in 8-bit mode at 2500 Hz frame rate, spatial resolution 157 nm, data collection rate 140 µm^2^ s^−1^. The scale bar in (*a*) indicates 2 µm. Reproduced from results reported by Jones *et al.* (2022 ▸).

Recently, at the XFM beamline of the Australian Synchrotron it was demonstrated that this dead-time-free operation permits flyscan ptychography with practically no overheads (Jones *et al.*, 2022 ▸). Detector overhead was eliminated with the continuous readout of the EIGER2 detector, whose two counters per threshold allow data to be buffered for readout during the acquisition of the next frame. This improved the quality of the image reconstruction, in the case where the amount of overlap was the same in the direction of the scan trajectory as perpendicular to it. Figs. 9 ▸(*a*) and 9(*b*) show two flyscan ptychography images of a test pattern [data from Jones *et al.* (2022 ▸)]: in (*a*) the EIGER2 detector was triggered externally at every point in the scan; in (*b*) the detector was operated in so-called ‘free-run’ software-triggered mode, starting each frame immediately after the end of the previous frame. The free-run acquisition shows an improvement in the spatial resolution, from 82 nm to 70 nm, as obtained from extracted line profiles across edge features in all directions (Jones, 2022 ▸). The frame rate of the detector was 50 Hz, resulting in a sampling interval of 100 nm, while the sample was continuously scanned at 5 µm s^−1^ velocity, which gives approximately 95% overlap of the 2 µm-wide beam between adjacent frames in both directions. These scans covered an area of 50 µm × 50 µm at a rate of 0.49 µm^2^ s^−1^, *i.e.* in around 85 min total scan time. However, based on statistical analysis of the diffraction data it was clear that the spatial resolution was not flux-limited. It was hypothesized, therefore, that the scanning speed could be increased with limited consequence to the reconstructed image quality.

The ability to immediately trigger a new frame with no dead-time permits increasing the frame rate substantially. Above 2250 Hz, and up to the maximum 4500 Hz frame rate, the EIGER2 X 1M detector dynamic range reduces to 8-bits (*cf.* ‘8-bit mode’ in Section 2.4.1), with the maximum count rate per pixel accordingly limited by the maximum counter value. For a high-speed scan in 8-bit mode, Jones *et al.* (2022 ▸) hence reduced the beam intensity by a factor of ten, and demonstrated ptychographic data acquisition at a 2500 Hz frame rate, while the sample was scanned at 250 µm s^−1^, yielding a similar 95% overlap in the scan direction, while decreasing the overlap in the non-scanning direction to 75%. This scan yielded a data collection rate of 140 µm^2^ s^−1^, covering a 440 µm × 800 µm area with a total scan time of 79 min. Fig. 9 ▸(*c*) shows the results from 8-bit mode high-speed scanning, with 157 nm spatial resolution for the same sub­region as shown in Figs. 9 ▸(*a*) and 9(*b*). For comparison, the scan in Fig. 9 ▸(*b*) covering an area of 50 µm × 50 µm at 0.49 µm^2^ s^−1^ would have required a scan time of around 20 s with these scan parameters. For the high speed scan, the X-ray dose was reduced by three orders of magnitude due to lower overlap in the direction perpendicular to the scan trajectory, and the ten-fold reduction of beam intensity. The ability to achieve super-resolution phase-contrast imaging with sub-millisecond framing time brings ptychography up to speed with X-ray fluorescence imaging, providing ultrastructural context to elemental and chemically specific information obtained simultaneously.

### 4.5. Single-pulse transient diffraction

Modern HPC detectors have the potential to exploit the pulsed nature of synchrotron sources without the need for additional beamline optics. Electronic gating allows individual X-ray pulses from a single bunch of the storage ring bunch structure to be isolated (Ejdrup *et al.*, 2009 ▸; Kraft *et al.*, 2009 ▸; Burian *et al.*, 2020 ▸; Bachiller-Perea *et al.*, 2020 ▸; Schmidt *et al.*, 2021 ▸). This approach allows for time-resolved experiments that are not limited by the readout speed of the detector, but only by the duration of the X-ray pulse, which is usually <100 ps (Burian *et al.*, 2020 ▸; Nakaye *et al.*, 2021 ▸). The temporal resolution in this time domain allows for investigations of ultrafast phenomena, such as light-induced reactions, phonon transport and transient structural dynamics (Cammarata *et al.*, 2008 ▸).

**Figure 10:**
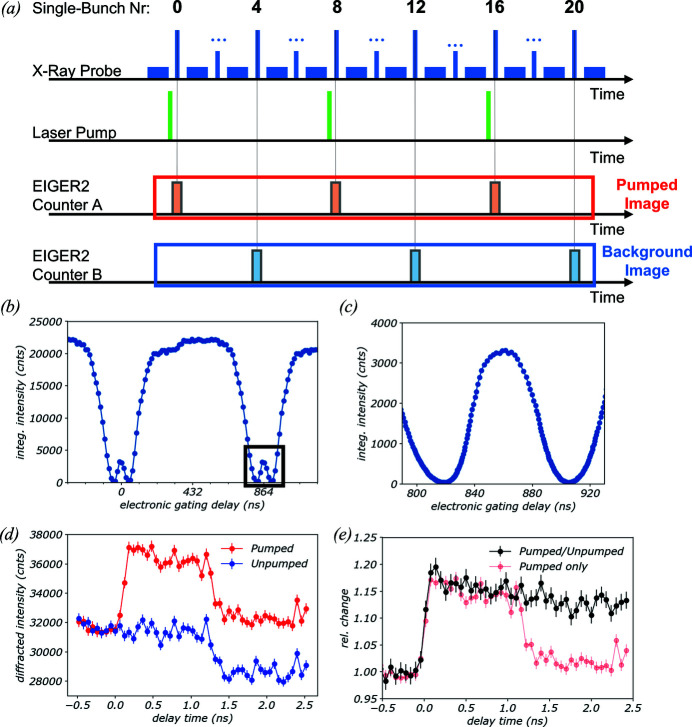
(*a*) Illustration of the timing scheme used in the experimental setup. The sample is excited every eighth ring repetition by an optical laser pump, while the detector is gated at double the frequency, so at every fourth ring repetition. In the double-gating mode, two consecutive gate signals alternately activate the two pixel counters (counters a and b) and simultaneously acquire images of the pumped and the background state. (*b*) Delay scan with the EIGER2 X 1M over the hybrid-mode filling pattern at the ELETTRA synchrotron. (*c*) High-resolution delay-scan of the detector gating signal around the single bunch, evidencing an effective gating duration of approximately 45 ns (FWHM). (*d*) Diffraction intensity of the zero-order GaAs/AlAs superlattice reflection in the proximity of the [002] AlAs peak as a function of pump–probe delay for the pumped (red) and unpumped (blue) images. (*e*) Relative transient diffraction intensity pumped/unpumped (black) compared with the relative pumped signal only (red), evidencing near-ideal background correction.

The Austrian SAXS beamline at the ELETTRA synchrotron recently established a flexible setup optimized for laser-pump/X-ray-probe experiments (Burian *et al.*, 2020 ▸). In this experiment the pump–probe setup was employed to investigate the heat conduction in a GaAs/AlAs semiconductor superlattice structure (Naumenko, 2023 ▸) using the EIGER2 double-gating mode (Section 2.4.1). A femtosecond laser (PHAROS; Light-Conversion, Lithuania) was phase-locked to the radio-frequency system of the ELETTRA storage ring, delivering optical pulses at 515 nm wavelength with a fluence of 3 mJ cm^−2^ at the sample plane. The laser beam [spot size 0.5 mm (H) × 0.5 mm (V)] was overlapped with the 8 keV X-ray beam [spot size 0.35 mm (H) × 0.1 mm (V)] which impeded the sample at a grazing angle of approximately 15.8° [effective X-ray spot size 0.35 mm (H) × 0.35 mm (V)], ensuring spatial homogeneity with regard to excitation and probing conditions (Reinhardt *et al.*, 2016 ▸; Burian *et al.*, 2020 ▸). X-rays were recorded with an EIGER2 X 1M in double-gating mode and with the ELETTRA storage ring operating in the ‘hybrid’ bunch-filling mode that offers a strong single bunch isolated from the continuous part of the filling pattern. An electronic delay generator (Highland-Electronics, USA) was phase-locked to the bunch frequency of the ELETTRA storage ring (1.157 MHz) and generating TTL-type gating signals (4.5 V) of 20 ns gate width at a quarter of that frequency (289.35 kHz). In this timing configuration [Fig. 10 ▸(*c*)], the detector isolates the radiation of the single bunch at every fourth repetition of the hybrid bunch structure. A delay scan of the detector gating signal over the storage-ring bunch structure in the hybrid filling mode [Fig. 10 ▸(*b*)] shows that the detector has an effective gating time of approximately 45 ns FWHM (depending on the energy threshold), and is hence capable of isolating the signal of the single bunch [see the high-resolution scan, Fig. 10 ▸(*c*)]. Jitter of the gating time over individual detector pixels could broaden the effective gating time. For the signal integrated from a very limited area of the detector of <10 pixels, here, no relevant contribution of jitter to the measured 45 ns is expected.

A pump–probe measurement (laser-pump/X-ray-probe) with stroboscopic acquisition was then performed, employing the EIGER2 double-gating mode. In this mode, every even-numbered X-ray pulse activates counter a, while every odd-numbered X-ray pulse activates counter b [*cf*. Fig. 2 ▸(*a*)]. By setting the laser repetition rate to half of the detector-gating frequency, *i.e.* to 144.675 kHz, the image collected in counter a records the optically excited state [Fig. 10 ▸(*a*), red gating pulses], while the other image, collected in counter b, acquires the unexcited state [Fig. 10 ▸(*a*), blue gating pulses]. This configuration hence brings the possibility for ideal background correction of the transient signal.

The benefit of the double-gating approach was investigated by studying the transient heat conduction in a GaAs/AlAs superlattice structure. We achieve this by measuring the strain-induced change in diffraction intensity of the superlattice zero-order reflection that originates from an intermixed GaAs/AlAs layered structure. The pumped image [red trace in Fig. 10 ▸(*d*)] shows a clear increase in diffraction intensity upon excitation (time delay: 0 ns), followed by an unexpected drop in scattering intensity at approximately 1.2 ns. The same drop of scattering intensity is also observed in the background image (blue trace), suggesting that this is likely the result of a ring injection in the ELETTRA top-up mode. By normalization, looking at the relative change in diffraction intensity as the ratio of pumped over unpumped signal in Fig. 10 ▸(*e*) (black trace), this drop in background signal can be corrected. This shows that the double-gating mode can significantly improve the data quality in pump–probe experiments, particularly compared with a typical single-gating acquisition scheme [light red trace in Fig. 10 ▸(*e*)].

## 5. Summary

Obtaining high-quality data and developing smart data collection strategies requires technical understanding of the experimental setup, including detectors. The first part of the paper clarifies technical aspects of EIGER2 detectors: performance, corrections and recently added features. The second part addresses how these technical aspects can be optimally used to develop methodologies and improve data quality at synchrotron sources.

## Figures and Tables

**Table 1:** EIGER2 parameters: ASIC and detector system

Pixel size (µm)	75 × 75
No. of pixels per ASIC	256 × 256
Maximum pixel count rate	>10 Mcounts s^−1^
Thresholds counters per pixel	2, low-energy discriminating 4 × 16-bit counter (2 per threshold)
Continuous readout	Yes (100 ns dead-time)
Bit depth of image output	32-, 16- or 8-bits
Module size (H × V)	77.1 mm × 38.4 mm (1028 × 512 pixels; 4 × 2 ASICs)
Special operating modes†	8-bit readout, Lines-ROI readout‡, double-gating
Sensor materials and thickness	Si 450 µm, CdTe 750 µm
Energy threshold range	Si: 3.5 keV (2.7 keV)‡ – 40 keV; CdTe 4 – 80 keV
Data output to detector control unit	Duplex fibre optic connections (4× 10 GbE per readout board)
Control interface	HTTP REST-like API
Data output interfaces/formats	Filewriter interface: HDF5; stream interface: ZeroMQ stream with header and data blob; monitor interface: TIFF
Data compression	BSLZ4 (default), LZ4

† Special operating modes have been implemented for detectors for synchrotron application; Lines-ROI mode for detectors comprising a single row of modules.

‡ The minimum threshold specified is 3.5 keV; it can be adjusted down to 2.7 keV by extrapolation.

**Table 2:** Data quality indicators for the standard 360° data collection as well as the Global Phasing Ltd workflow

	Single orientation, 360°	GPhL workflow: three orientations, 2 × 210° and 1 × 360°
Molecular weight of asymmetric unit (kDa)	560	560
Diffraction limits (Å) along a*, b*, c*	1.14, 1.07, 1.22	1.08, 1.09, 1.05
*R*_merge_ / *R*_pim_	0.132 / 0.037	0.123 / 0.027
Total reflections	25980668	49847294
Unique reflections	1892149	2320166
Completeness (%)	95.6	97.6
Mean *I*/σ(*I*)	12.8	16.1
Multiplicity	13.7	21.5

## Appendix A HPC detector performance parameters

This appendix describes common detector performance parameters in the context of hybrid-photon-counting detectors.

### A1. Noise and background-free detection

Unlike integrating detectors, HPC detectors are free of noise associated with the read-out operation and signal offsets (detector background). Leakage signals that would cause background and noise on integrating detectors are fully suppressed by the energy threshold. This allows the accuracy of the measurements to reach the fundamental noise limit of the X-ray counting process, determined by the variation on the number of counted X-ray photons described by Poissonian statistics. The remaining detector background is ‘real’ and determined by the natural radiation background (compare background measurement for EIGER2 presented in Appendix *B* [App appb]).

### A2. Dynamic range and bit depth

By suppressing the electronic background signal and noise, HPC detectors can simultaneously detect weak and strong X-ray signals, *i.e.* covering a wide intensity range of intensities in one image. The dynamic range (DR) of an X-ray detector is defined as the ratio of the ‘maximum signal within the linear range’ to the ‘minimum detectable signal’ (Ponchut, 2006 ▸): DR = *S*_max_/*S*_min_. The minimum detectable signal for a photon-counting detector is *S*_min_ = 1 count (one photon). Assuming the incoming intensity is within the linear regime of the count-rate range, the detector will register a count for each detected photon until the counter is full, *i.e.* as long as the pixel counter is large enough for the number of photons to be detected. The dynamic range of a photon-counting detector is thus given by the maximum number of counts that can be acquired in a single exposure, given by the maximum counter value. However, due to the absence of detector background and noise, with HPC detectors it is possible to split up long exposures into several short exposures, which can be summed to circumvent the counter-depth limit completely (*cf*. Section 2.4.1 on auto-summation).

The maximum counter value, and also the dynamic range, is often described by the number of bits of the counter, its bit depth *n*, with maximum counter value 2^*n*^ − 1.

### A3. Quantum efficiency

The QE is defined by the following ratio: QE = (No. of counted photons)/(No. of impinging photons). It depends on the sensors’ material and thickness.

Related specifications are the absorption efficiency and the detective quantum efficiency (DQE). Absorption efficiency describes the fraction of photons absorbed by the sensor (Beer–Lambert law), and the DQE describes the degradation of the signal-to-noise ratio of the incoming signal (X-ray image) by the detector.

### A4. Spatial resolution – point spread function

The point spread function (PSF) describes the blurring added by the detector system to an incoming X-ray image. Due to the direct detection, the charges created by an absorbed X-ray inside the sensor are collected mostly by the readout pixel below the detection event. Even for detection events at the pixel borders, in which charge is shared between signal inputs of neighbouring pixels, the closest pixel will collect the majority of charges. With a threshold setting of 50% of the photon energy, only the pixel closest to the absorption event will count the event, as the remaining charge is <50% of the total and too low to trigger another pixel. This resulting PSF is therefore sharply defined by the pixel geometry, with a FWHM approximately equal to the pixel pitch (Zambon *et al.*, 2019 ▸).

## Appendix B Background count rate of EIGER2

Due to the absence of detector-generated background, even the natural high-energy background radiation from cosmic radiation and natural radioactivity becomes visible on HPC detectors. Although it is low intensity, this natural background may be limiting low-flux applications that require long exposure times.

Measurements performed on EIGER2 Si and CdTe detectors using the lowest specified threshold energy (3.5 keV and 4.0 keV, respectively) and disabled instant retrigger show a natural radiation background as low as ∼0.07 counts h^−1^ pixel^−1^ for Si detectors and ∼0.4 counts h^−1^ pixel^−1^ for CdTe detectors. The difference between Si and CdTe here is attributed to the higher stopping power for high-energy radiation of the 0.75 mm CdTe sensor compared with the 0.45 mm silicon sensor. The values were determined from the analysis of over 100 h of dark images (images without X-ray exposure) recorded during the detector burn-in process at the DECTRIS Ltd factory. However, the exact background count rate depends on the local environment.

In order to suppress high-energy background even further, the second detector threshold on EIGER2 can be used as an upper energy discriminator to detect and suppress high-energy events efficiently by using the detector’s difference mode (as for high-resolution PXRD in Section 4.2.1) or by individual treatment of detection events and using a second upper threshold (Baron & Ishikawa, 2023 ▸).

## Appendix C Quality indicators for MX data in Section 4.1

Table 2 ▸ summarises the data quality indicators for the standard 360° data collection as well as the Global Phasing Ltd workflow. Note that the total dose to the crystal amounts to 1.5 MGy in both data collections (Chari *et al.*, 2023 ▸).
